# Health and Social Sciences working together in Community-Based Learning: Is this possible?

**DOI:** 10.15694/mep.2017.000008

**Published:** 2017-01-18

**Authors:** Leide Da Conceição Sanches, Leandro Rozin, Izabel Cristina Meister Martins Coelho, Patricia Helena Napolitano, Christiane Luiza Santos, Elaine Rossi Ribeiro

**Affiliations:** 1 Faculdades Pequeno Principe

**Keywords:** MEDICAL EDUCATION, SOCIAL SCIENCES, COMMUNITY BASED LEARNING

## Abstract

This article was migrated. The article was marked as recommended.

This is a case study of the experience of integration of health teaching and the community, which aims to bring medical students closer to the context in which health users are inserted. This paper aims to present a case related to the experience of a multiprofessional team in the Community Based Learning Module of an undergraduate course of Medicine in the State of Paraná/Brazil, the goal of which is to train physicians with a solid technical and scientific profile based on a holistic vision that allows a competent professional action on the health and disease process in the perspective of health care integrality, with social responsibility and commitment to citizenship. The case presented shows that it is possible to use methodologies favoring the active participation of the student in the construction of knowledge and integration of contents; promotion of integration and interdisciplinarity that is consistent with the axis of curriculum development, seeking to integrate the biological, psychological, social and environmental dimensions.

## Community Based Learning Module: Timeline

The organization of the module takes place weekly with meetings of the Planning Group, when relevant points of the teaching-learning process are discussed with the multiprofessional teaching staff. The 50 students in each class (2 classes per year) are divided into eight groups, where four groups go to the practice activities in the Health Units accompanied by the teachers, and the other four groups stay in the classroom for study and theoretical reflection, using Maguerez arc (apud Bordenave, 1982), related to the practice activity performed in the previous week.

"Update Activities" are also programmed in the classroom, the focus of which is to approach the theory with the use of active methods of health teaching, the themes proposed in the learning objectives of the semester. Thus, the student has a theoretical background, and practice experiences in the Health Unit.

## Community Based Learning Module I: Health and Society

CBL I, the theme of which is Health and Society, represents the initial insertion of the student in the community, and so covers the development of bond among teachers, services and the community, with broader concepts about society, stemming from the understanding of the human being in the social, anthropological and ecological dimensions for the determination of the health-disease process, with an emphasis on the comprehension of the relations between health and society organization. When inventorying the social equipment of the health care network of a territory, and its influence in the promotion and recovery of health, the student comes to understand that being a health professional presupposes a broad knowledge of society and of the human being in his/her entirety.

What emerges in CBL I is an increasingly effective approximation of medical students to the community, and the notion of the social role of the physician in face of the diversity of determinations of the health-disease process, be they cultural, behavioral, psychological, ecological, ethical and legal, at individual and collective levels. This report shows that the experience of both medical students and CBL teachers has developed an important link of the teachers, the health services, and the local community. The Health Units have become an important area of sociability, where our teachers and students get closer, not only to the users, but to their families and the teams of professionals who accompany them. In addition, the format of the practical activities allows the students to follow the same community, which allows the deepening of the social relations that are established, as well as more commitment and responsibility of the teachers and students of the medical course with the community.

The continuous interaction leads to an inevitable approximation to the community, and consequently the problems appear and stand out to the teachers and students, who would not perceive them if they were only passing through. It is the approach established by the planned routine of activities that allows an approximation with the daily practices in the areas visited. It is in the space of sociability of daily life of users of the Health System that the broadening of the worldview becomes possible, allowing to understand the meanings of health promotion. Besides the enrichment of the exchange of experiences, the space of sociability of the Health Units promotes growth of both the students involved and the teachers, with considerable impact for their growth and maturity. At the end of CBL I, students develop an activity called Diversity Exhibition: one Brazil, many nations. The students were able to identify cultural differences and the real meaning of health for each ethnic group. Besides that, the students take attitudes of a professional-to be in the future, where they could find ethnic differences during the process of diagnosing, treating, promoting and rehabilitating in health. The presented pedagogical action showed that the student runs after realistic data, showing both the beauty and the stigmas of each ethnic group, promoting a very playful and consistent learning.

## Community Based Learning Module II: Health Systems

CBL II, the theme of which is Health Systems, studies the historical process of construction of the Unified Health System in Brazil, with an analysis of the components for formulating social and health policies. Among them, there are the health care models and the organization of Primary Health Care as its structuring component, with the appropriation of the territory through historical, demographic, socioeconomic, environmental and epidemiological aspects of the community, and of the health services of the area covered by the Health Unit.

The structuring axis of the period is the construction of the Community Diagnosis (DC), elaborated by the students based on the practical activities in the Health Units (US). The DC is a guiding tool for health work that has been effective in achieving the learning objectives of the module, because it identifies the problems and needs for resources by the communities.

The Community Diagnosis provides extremely important information, which guides the real situation of the community, in a broad or specific way, and supports the health teams to define care priorities that result in the promotion of health and the prevention of diseases. In addition, the Community Diagnosis is used to identify health risks and diseases in different age groups, describes a reality that can be studied for the application of health actions and programs, and provides the basis of priorities according to the information identified (DUNCAN, SCHIMIDT E GIUGLIANI, 2004).

In order to perform a DC, it is necessary to know the health policies, health surveillance, primary health, health and society (anthropology and sociology), addressed in CBL I. Students get data available in health information systems, transform these data in health indicators, prioritizing chronic communicable and non-communicable diseases, with socio-demographic distribution.

The research and construction of the DC generates a final seminar that the groups of students, who remain in different US practices, present to the faculty, colleagues and managers of these units.

## Community Based Learning Module III: Manangement and Public Policies

CBL III, the theme of which is Management and Public Policies, seeks the training of the physician on the principles, guidelines and policies of health systems, so that the future professional can participate in management and administration actions, both in the public and private healthcare network and thus, promote the community's well-being (BRAZIL, 2014). It recognizes the importance of a participatory construction of the health system, to understand the role of citizens, managers, workers, and instances of social control in the elaboration of the Brazilian health policy (BRASIL, 2014).

Based on conceptions about health and illness, and its influences on the demand/supply relationship of health services, the students articulate the previous knowledge about the use of information systems, and produce an analysis that stems from the identification, explanation of health problems, and ways of collective coping with problems. To do this, they apprehend elements of health service management, such as planning, monitoring, evaluation and financing.

During the semester, the students have contact with managers of different levels of attention of both public and private services, either through technical visits or updating activities. In addition, students make an incursion into primary health care services to investigate how they are organized and managed.

With this, they are inserted in the practice settings in order to identify the activities, structures and internal organization of the health services in the first level of attention, both to the assistance of the spontaneous demand, and the forms of attendance of the organized offer (programs, protocols, and care networks), and they are monitored and evaluated. They also seek to understand the relationship between primary care and the other levels of care of the Municipal and Regional Health System, and to know what the challenges for comprehensive and universal health care are.

As a final product, the students present a situational analysis of the Primary Care Units management processes in which they were inserted, and individually produce a reflexive, critical portfolio about the experiences they lived.

It is also worth mentioning that in view of the intentionality of all CBL modules to work in close approximation with the thematic modules of the current semester, in this CBL module the construction of this analysis on the management processes takes place in a more directed, but not exclusive way, on the mother and child care network, the rehabilitation network, and the oncology network.

## Community Based Learning Module IV: Family Health Strategy

In CBL IV, the theme is Family Health Strategy, and the summary refers to the discussion of the historical and legal philosophical bases of the Family Health Strategy, and the concepts of Family and Community Physician (MFC); the principles and characteristics of the physician and staff work in the Family Health Strategy/Primary Health Care, with discussions about the prominent national and international experiences on Family Medicine. In practical activities, the theoretical and practical relationship with the person, the family and the community takes place, and is based on the main elements of the various life stages, namely: child health fundamentals, adolescent health, women's health, adult health, and elderly health within Primary Health Care. The students are aware of problem-oriented registration, telehealth, and access to Primary Health Care, Quaternary prevention, and evidence-based medicine.

During this period, the students make home visits with another look, viewing the context of the health-disease process, with an approximation of clinics, diagnosis and treatment. Another subject that also permeates the module in a transversal way is Primary Health Care, and the discussion of complex cases used as part of the methodology.

The learning objectives are: to identify and know participative management and health practices, in the form of teamwork, and the forms of registration and follow-up of the ESF at the US; to know the characteristics of the ESF in the city of Curitiba, and to distinguish them from other experiences; to know and analyze the positive results that ESF has produced in the main health indicators, and in the reorganization of the attention model, with the search for greater rationality by the other levels.

The module is formulated to meet the principles of the European Society of Family and Community Medicine, in search for the development of core competencies, namely: Management of primary health care, person-centered care; specific problem-solving skills; comprehensive approach; community orientation and holistic modeling (WONCA, 2002)

Finally, to contribute to a closer approximation of contents, the students prepare and present some real cases in the form of an "Exhibition", always relating them to the themes studied in the thematic modules, and experienced in the practice activities at the Health Units.

## Community Based Learning Module V: Family Approach

In CBL V, the central theme is the Family Approach. Initially, the concepts of the Person-Centered Clinical Approach are reinforced, with the comprehension that this is critical in the physician-patient relationship. Regarding family care, the longitudinal and holistic follow-up is searched, through Family Medicine tools in the approach at home, still with the approach of anthropology and medical sociology. The purpose is to broaden the clinical view, leaving the doctor's office, and checking the importance of the family view on the health-disease process.

The concept of family, its legal and historical aspects and contemporary family arrangements are deepened, preparing the student to cope with diversity and complexity of the context and the community. The communities where the activities are carried out are of low income. One of the topics dealt with the students is the Child and Adolescent Statute (ECA), considering the type of demand and referral to be made. They also make 4 home visits, with 2 families for each group of 6 students.

In this learning setting, the students are led to develop their communication and management competency with the US health team, because it is the family doctors and health agents who indicate the families to be visited, and who demand a return with a written report to the Unit, as well as the family genogram. During the visit, the students make an interview, establish bond, get closer to the various family members, and use family medicine tools: Genogram, Practice, FIRO and Ecomap. 

The view of diversity of knowledge areas is a differential of the course since the first year. The students absorb the difference very well. The goal is to understand the complexity of the family system, the specificity of the disease, the possibilities that the Health System sometimes does not view; far from being the diagnosis or prognosis of the disease, it is rather the strengthening of health and healthy processes. The family is guided in this direction, in the search for health at all levels.

The students' reports at the end of the semester revealed that: "we now have better conditions to perform ambulatory care with much more attention to the context in which the patient is inserted. This context makes all the difference."

The family, who is previously visited in the CBL IV, receives home visits in the fifth period, and will be followed up again in the US in the sixth period, thus fulfilling the longitudinal aspect of patient follow-up, allowing the establishment of bond, and the experience of process advances and setbacks.

As a final product, each team produces a film of up to 5-minute duration, with a high degree of excellence. The films showed: sensitivity to the family context, pain, disease process, "cry" for help through the sickness of the Identified Patient, practical application of the tools, and the understanding of the concepts and principles of Family Medicine.

## Community Based Learning Module Evaluation

The evaluation dimension of CBL module comprises the student's formative and summative learning evaluation. It is understood as a continuous and systematic process of monitoring and judgment of the level at which students are, regarding the achievement of established goals and expected performances. Cognitive/summative assessment of students is done through a written test with multiple-choice questions. The formative evaluation includes feedback on all practice activities and portfolio.

For the evaluations of the portfolio, final seminar, and practices carried out in the Health Units, instruments were created with criteria such as: theoretical and practical considerations and critical reflections of the subjects studied, which were widely debated by the team of teachers of the CBL modules, and later approved by the Commission for Student and Course Assessment (CAEC).

## Final Considerations

Thus, the purpose of CBL is to early integrate the student in the community, and promote the student's approach to the social role of the physician in face of the cultural, ecological, psychological, biological and economic determinations of the health and illness process. It has peculiar characteristics, and it is configured as longitudinal, since it is kept in the curriculum for 8 consecutive periods. Currently, the course that started with the first group in the second semester of 2014 has worked so far from CBL I to CBLV. It is thought that in the future CBL VI, with the theme Primary Care Management, CBL VII, with the theme Education In Health, and CBL VIII with the topic of Clinical Management, can be equally disseminated after their implementation.

In this case study, it is concluded that it is the approach established by the planned routine of activities that allows an approximation with daily health practices. It is in the space of sociability of the Health System users' daily life that the worldview broadening becomes possible, for the comprehension of meanings of health promotion to take place. Ribeiro (2016) says that in addition to the exchange of experiences, the space of sociability of the Health Units provides growth for all those involved. Health promotion occurs as a result of this maturity of both teachers and students, in the approximation of the reality of health services and the community. 

## Take Home Messages

- It is possible to integrate social sciences and community based learning.
- The worldview changes when there is social reflexions.
- The space of sociability in practical activities provides growth for all those involved.

## Notes On Contributors

Elaine Rossi Ribeiro

Graduate at Nursing at Universidade Estadual de Londrina - UEL. Master in Education at UEL. PHD and Msc in Surgery from UFPR - Federal University of Paraná. Currently, she is Academic Director of the Institute of Education and Research - IEP / FEAES and researcher professor in the Master of Health Professions Educations at Hospital Pequeno Príncipe.

Leide Da Conceição Sanches

Graduated in Social Sciences (1993) and graduated in Law from Pontifical Catholic University of Paraná (1998). PhD and MSc in Sociology from the Federal University of Paraná - UFPR. She is currently a professor of Sociology and Anthropology Applied to Health, member of the Research Group on Health Sociology UFPR and Coordinator of the Research Ethics Committee of Hospital Pequeno Príncipe.

Leandro Rozin

Bachelor's Degree in nursing at Faculdades Pato Branco, Master's Degree in Biotechnology Applied to the Health of Children and Adolescents at Hospital Pequeno Príncipe. Professor in several disciplines in health science courses at Faculdades Pequeno Príncipe. Member of Core Nursing Course Structuring Teacher.

Patricia Helena Napolitano

Graduated in Psychology from the State University of Londrina (1986) and Master in Education from the State University of Londrina (1997). She acts mainly in the subjects: psychology, education, low-income community, psychotherapy, family therapy, interpersonal development, loss and mourning. Professor at Faculdades Pequeno Principe.

Christiane Luiza Santos

Graduated in Dentistry from UFPR. Master in Public Policies by UFPR. Currently studying for a PhD in Public Policies at the Federal University of Paraná. He works with Public Health in the areas of Primary Health Care, Territorialization, Health Management, Health Work Process Assessment, Public Policies, Permanent Education, Health Promotion and Prevention. Teacher at Faculdades Pequeno Principe.

Izabel Cristina Meister Martins Coelho

Medical Doctor by UFPR - Federal University of Paraná, Master in Surgical Clinics and PhD in Clinical Surgery at the UFPR.She's surgeon in Liver Transplant at Hospital Pequeno Principe and in Emergency Unit of the UFPR. Currently, she coordinates the Medical School and the Master of Health Professions Educations in Hospital Pequeno Príncipe.

## Bibliography/References

Berbel, N. A. N. Metodologia da Problematização no Ensino Superior e sua contribuição para o plano da praxis. Semina: v.17, n. esp., p.7-17, 1996. 

Bordenave, J; Pereira, A. Estratégias de ensino aprendizagem. 4. ed., Petrópolis: Vozes, 1982. 

Brasil. Ministério da Educação. Conselho Nacional de Educação. Resolução nº 3, de 20 de junho de 2014. Institui Diretrizes Curriculares Nacionais do Curso de Graduação em Medicina e dá outras providências. Brasília. 2014. 

Canesqui, Ana Maria. Sobre a presença das ciências sociais e humanas na saúde pública. Saude soc., São Paulo, v. 20, n. 1, p. 16-21, March 2011. Available from <http://www.scielo.br/scielo.php?script=sci_arttext&pid=S0104-12902011000100003&lng=en&nrm=iso>. accessed on 20 Dec. 2016.

https://doi.org/10.1590/S0104-12902011000100003

Duncan, B.B, Schimidt, M.I., & Giugliani, E.R.J. Medicina ambulatorial: condutas de atenção primária baseada em evidências. 3ed. Porto Alegre: Artmed.2004. 

Gallagher, S. Wallace, S. Nathan, Y. & McGrath, D. 'Soft and fluffy': medical students' attitudes towards psychology in medical education. Journal of Health Psychology 20 (1): 91-101. 2013

https://doi.org/10.1177/1359105313499780

Luz, M. T. Políticas de Descentralização e Cidadania: novas práticas de saúde no Brasil atual. In: Pinheiro, R.; Mattos, R. A. (Organizadores). Os sentidos da integralidade na atenção e nos cuidados à saúde. 8ª ed. Rio de Janeiro: Abertura edição, 2009. 

Ribeiro, E.R. et al. Integration community based learning: looking for health promotion. Amee Abstract Book.2016. 

Wonca. A definição europeia de Medicina Geral e Familiar (clínica geral / medicina familiar). Organização Mundial da Saúde. Barcelona, 2002.

## Appendices

## Declarations

The author has declared that there are no conflicts of interest.

