# Supplementary material for: Health and Social Sciences working together in Community-Based Learning: Is this possible?
Source: MedEdPublish (2016). 2017 Jan 18;6:8. [Version 1] doi: 10.15694/mep.2017.000008 (PMC10885216; doi:10.15694/mep.2017.000008)
Supplement: Peer review history

# Reviewer report: Reviewer response for version 1

**DOI:** 10.21956/mep.19420.r28529

**Published:** 2018-04-27

**Authors:** Anthony David.M

**Affiliations:** 1 Soho

This review has been migrated. The reviewer awarded 4 stars out of 5 An interesting article indeed! The longitudinal integration of Community Health with the teaching of medicine is very much work-based approach.Similar such experiments have been done in developing nations like Brazil which were termed Community Orientation Programs going on every professional year of the five year course in my alma mater Christian Medical College Vellore, India. I am a product of such 'immersion experiments" and must admit that they helped me to get a community point of view. The Brazilian experiment seems to be more quantifiable and can be replicated and studied in other environs.Congratulations authors!

Reviewer Expertise:

NA

No decision status is available

# Reviewer report: Reviewer response for version 1

**DOI:** 10.21956/mep.19420.r28528

**Published:** 2017-02-17

**Authors:** P Ravi Shankar

**Affiliations:** 1 American International Medical University

This review has been migrated. The reviewer awarded 4 stars out of 5 Brazil is among the leaders in integrating health professions education with health systems. Thus I found it interesting to read this manuscript describing an initiative from the country. The authors can provide a brief introduction to the medical school and it is integrated with healthcare delivery in the state at the beginning of the Introduction. The authors have provided a detailed and comprehensive description of the various community-based learning modules being conducted at their institution. As mentioned by the authors, repeated visits to the same community and families throughout their course strengthens the bonds between the students and the community. Though as pointed out by another reviewer there may be some issues with the use of language in this paper, this is an important initiative and the authors should be congratulated for their efforts. The authors can provide more details about how the module is evaluated but this I believe may their focus in another paper.

Reviewer Expertise:

NA

No decision status is available

# Reviewer report: Reviewer response for version 1

**DOI:** 10.21956/mep.19420.r28527

**Published:** 2017-02-17

**Authors:** Luiz Ramos

**Affiliations:** 1 Up

This review has been migrated. The reviewer awarded 5 stars out of 5 Interesting article.It's very important that the students have this kind of practice and be able to relate the knowledge they learn at college and the work at field with the community. Congrats to the authors.

Reviewer Expertise:

NA

No decision status is available

# Reviewer report: Reviewer response for version 1

**DOI:** 10.21956/mep.19420.r28535

**Published:** 2017-02-16

**Authors:** João paulo João

**Affiliations:** 1 Life

This review has been migrated. The reviewer awarded 5 stars out of 5 Great articule!

Reviewer Expertise:

NA

No decision status is available

# Reviewer report: Reviewer response for version 1

**DOI:** 10.21956/mep.19420.r28530

**Published:** 2017-02-16

**Authors:** Kendra Thomas

**Affiliations:** 1 University of Indianapolis

This review has been migrated. The reviewer awarded 5 stars out of 5 Congrats to the authors and contributors for their interdisciplinary work and service to the field!

Reviewer Expertise:

NA

No decision status is available

# Reviewer report: Reviewer response for version 1

**DOI:** 10.21956/mep.19420.r28534

**Published:** 2017-02-09

**Authors:** Elisangela de Mattos e Silva

**Affiliations:** 1 faculdades Pequeno Principe

This review has been migrated. The reviewer awarded 5 stars out of 5 It's an excellent and very interesting article with useful and very important contribution to all medical students, teachers and institutions

Reviewer Expertise:

NA

No decision status is available

# Reviewer report: Reviewer response for version 1

**DOI:** 10.21956/mep.19420.r28532

**Published:** 2017-02-03

**Authors:** elisie costa

**Affiliations:** 1 pmc curitiba

This review has been migrated. The reviewer awarded 5 stars out of 5 This article comes to teach Us many important things about health no Brazil, The experience with this methodogy must be used in other places to improve health education

Reviewer Expertise:

NA

No decision status is available

# Reviewer report: Reviewer response for version 1

**DOI:** 10.21956/mep.19420.r28533

**Published:** 2017-01-27

**Authors:** Isabel de Lima Zanata

**Affiliations:** 1 Institute of Education and Research - IEP / FEAES

This review has been migrated. The reviewer awarded 5 stars out of 5 This article is a great contribution to the area of ​​medical education. It is interesting the reflection that the article does about the integration of health teaching and the community. It presents a new teaching strategy for medical students, bringing them closer to practice in public health in Brazil. I suggest that the authors write more papers on this subject, describing other methodologies that may be used in medical education.

Reviewer Expertise:

NA

No decision status is available

# Reviewer report: Reviewer response for version 1

**DOI:** 10.21956/mep.19420.r28531

**Published:** 2017-01-18

**Authors:** Trevor Gibbs

**Affiliations:** 1 AMEE

This review has been migrated. The reviewer awarded 3 stars out of 5 Although I found this paper a little initially difficult to understand because of the language issues, after several iterations I began to understand and realised that it relates to an important innovation within Brazil in providing a Longitudinal Integrated Curriculum activity that addresses the teaching element of a Socially Accountable school. The various learning outcomes described were very appropriate for integrating a community-engaged curriculum with public health and social medicine. I would have liked to have learned more regarding the teaching methodologies used and who were the teachers that delivered this programme.For the evaluation section, I would have looked more to reading about the product- how this had changed the students and the community they worked in- at the moment it reads more as an innovation that takes from the community, rather than one that gives back to the community.I wish the authors well in developing this programme and look forward to hearing more from them in the future

Reviewer Expertise:

NA

No decision status is available
